# Ultra high-resolution biomechanics suggest that substructures within insect mechanosensors decisively affect their sensitivity

**DOI:** 10.1098/rsif.2022.0102

**Published:** 2022-05-04

**Authors:** Gesa F. Dinges, Till Bockemühl, Francesco Iacoviello, Paul R. Shearing, Ansgar Büschges, Alexander Blanke

**Affiliations:** ^1^ Institute of Zoology, University of Cologne, 50674 Cologne, Germany; ^2^ Electrochemical Innovation Lab, Department of Chemical Engineering, University College London, WC1DE 6BT London, UK; ^3^ Institute of Evolutionary Biology and Ecology, University of Bonn, 53121 Bonn, Germany

**Keywords:** load sensors, campaniform sensilla, finite-element analysis, principal component analysis, *Drosophila melanogaster*, strain

## Abstract

Insect load sensors, called campaniform sensilla (CS), measure strain changes within the cuticle of appendages. This mechanotransduction provides the neuromuscular system with feedback for posture and locomotion. Owing to their diverse morphology and arrangement, CS can encode different strain directions. We used nano-computed tomography and finite-element analysis to investigate how different CS morphologies within one location—the femoral CS field of the leg in the fruit fly *Drosophila*—interact under load. By investigating the influence of CS substructures' material properties during simulated limb displacement with naturalistic forces, we could show that CS substructures (i.e. socket and collar) influence strain distribution throughout the whole CS field. Altered socket and collar elastic moduli resulted in 5% relative differences in displacement, and the artificial removal of all sockets caused differences greater than 20% in cap displacement. Apparently, CS sockets support the distribution of distal strain to more proximal CS, while collars alter CS displacement more locally. Harder sockets can increase or decrease CS displacement depending on sensor location. Furthermore, high-resolution imaging revealed that sockets are interconnected in subcuticular rows. In summary, the sensitivity of individual CS is dependent on the configuration of other CS and their substructures.

## 1. Introduction

Campaniform sensilla (CS) are load sensors, embedded near joints within the exoskeleton of insect legs, that measure tonic forces and their rates of change [1–4]. Each CS consists of cuticular substructures, such as a cap, collar and socket, and multiple internal cell bodies including a neuron [5,6]. The deformation of the cuticular substructures is a crucial aspect of mechanotransduction [6,7]. Furthermore, the relative position, eccentricity and orientation of the cap enable directional sensitivity when transducing cuticular compressions induced by external and muscle-generated forces [8–11]. Sensory discharge from CS informs neuro-muscular networks on the limb's state, especially regarding load. This proprioceptive feedback can help modify and reinforce motor output in a task-dependent manner, ultimately enabling adaptable movements, such as walking [12–15].

**Figure 1.**
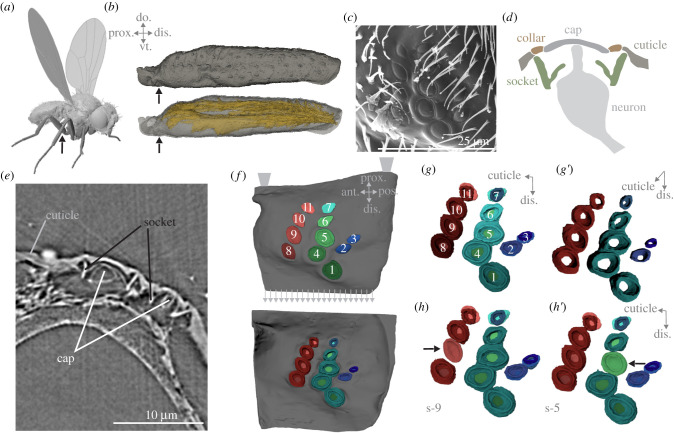
Location and arrangement of the CS field and finite-element simulation set-up. (*a*) Three-dimensional rendered fly, the arrow indicates the location of the femoral sensilla field (FeF) of the metathoracic leg; (*b*) three-dimensional rendered images of the femur, indicating the location of the FeF. Femur is shown in two different opacities to underline the proximity of the FeF to the femoral muscles; (*c*) scanning electron microscopy image of the metathoracic FeF. Image modified from Dinges *et al*. [16], scale bar 25 µm; (*d*) schematic of a CS; (*e*) nano-CT slice of the FeF; (*f*) nano-CT-based segmentation of the FeF. The colours and numbers of each cap are used throughout the publication, trapezoids indicate model constraints, arrows indicate forces applied; (*g*) the sockets underneath the caps seen in (*f*), viewpoint from the inside of the leg (i.e. distal end of the socket); (*g′*) the sockets underneath the caps seen in (*f*), viewpoint from the cuticle inwards (i.e. proximal end of the socket); (*h*) the set-up s-9, with the socket of CS 9 removed (indicated with an arrow); (*h′*) the set-up s-5 with the socket of CS 5 removed; do: dorsal, vt: ventral, prox: proximal, dis: distal, ant: anterior, pos: posterior.

CS are usually found in close proximity to joints (figure 1*a,b*); there, the sensor substructures work together to generate sensory discharge in response to cuticle bending. The cap of a CS is suspended over a hole in the cuticle and is attached to it via the collar and the joint membrane [17–19]. Below the cap, a bipolar neuron's modified dendrite reaches the cap's underside, while the axon of the neuron projects into the ventral nerve cord (morphology schematized in figure 1*d*). The dendrite is surrounded by a socket [20]. Furthermore, collars can act as stiffening rings that alter a caps sensitivity to the global strain [21,22]. However, not all *Drosophila* leg CS have visible collars (figure 1*c*), suggesting intra-group strain alterations that may not affect all cap displacements in the same way [16].

The geometry of CS substructures directly affects how CS transduce strain into action potentials. Compressive strain displaces the cap vertically by compressing the hole underneath the cap horizontally. Because the sensory neuron's dendrite is connected to the cap, it elongates along its longitudinal axis [6]. Through the orientation of CS with varying degrees of eccentricity (figure 1*c*,*f*), the sensitivity of individual CS to strains arising from distinct directions is modulated. CS with higher eccentricities (*elliptical*) compress along their short axis [15]. Additionally, CS with an eccentricity of zero (*circular*) are also present on the legs (figure 1*c*,*f*; [16,23]) and can either be directionally sensitive through asymmetric collars or act as omnidirectionally sensitive tactile sensors [10,15]. The grouping of diverse CS morphologies can provide the nervous system with highly specific information on local strain directions and magnitude [11,21]. CS are often found in clusters (i.e. groups or fields) and sometimes as single CS. Single CS may function as general strain indicators; multiple CS, with the same cap orientations but differing eccentricities, can form functional units when aligned, which can compute sophisticated features of load signals (e.g. by fractionating strain; [1,24–26]).

The structural components of each CS may influence the compressive strains sensed by surrounding CS, but it is unclear how CS substructures interact and affect strain proliferation. Modelling studies have shown that multiple holes in a structure, like an exoskeleton, can alter the overall compliance at that location [21,22,27]. Although CS themselves do not affect the cuticle's stiffness, the cuticular holes amplify local stress when a force is applied [21,22,27]. This indicates that CS arrangement in fields may have specific amplification functions that can influence the mechanical filtering of sensory input [20]. Further, CS morphologies within a field range in eccentricity, size and relative orientation (figure 1*c* as an example). In the drosopholid leg, there are two CS fields. The femoral CS field contains a variety of morphologies in proximity, which may imply that individual CS sensitivities are influenced through the interplay of all sensors (all CS locations can be seen in [16] and [23]).

We investigated strain distribution in this CS field, which contains sensors varying in size, orientation and morphology, as well as differences in key aspects, such as the presence of a strain-amplifying collar. The differences seen in this field may modulate strain, creating amplifications for individual CS induced by others. Using nano-computed tomography (nano-CT), we constructed a finite-element analysis and identified further morphological attributes of the field, such as sockets that are connected to one another, ultimately creating groups within the field. The finite-element analysis was used to investigate how physical connections between CS, which could influence strain distribution, and different CS parameters, which can alter the sensitivity, affect the displacement. Thus, CS parameters affect the displacement throughout the field upon the application of stress.

Previous investigations have shown how structural components of CS contribute to mechanotransduction. Skordos *et al*. [22], modelling a single CS, found that the stiffness of the septum, cap and cuticle does not affect transduction while the collar's material properties can tune the sensitivity of a sensillum. Vincent *et al*. [27] modelled a CS field as ‘holes’ in the cuticle and thereby showed that greater sensitivity is achieved when these holes are arranged in a regular pattern. Additionally, an evenly spaced pattern increases the energy absorption of the cuticle, which amplifies the strain around each sensillum. Kaliyamoorthy *et al*. [28] combined finite-element analysis and confocal microscopic images of the cockroach trochanteral CS to analyse how modelled climbing compresses different CS fields. By using a high-resolution physiological CS field in a finite-element analysis, we expanded on these models by showing that the previously uncharacterized morphology of CS substructures influences strain dynamics.

The present study advances current knowledge about the mechanosensation of CS by combining accurate morphological data with naturalistic force data extracted from jumping flies to infer the influence of CS substructures on strain magnitudes and distributions. In conclusion, our model connects high-resolution CS morphologies with realistic strain in the leg of *D. melanogaster* and provides a basis for future kinematic models.

## 2. Material and methods

### 2.1. Sample preparation and data acquisition

The metathoracic legs of female wild-type *D. melanogaster* (Berlin-K, RRID:BDSC_8522) aged 3–5 days post-eclosion were fixed using 4% paraformaldehyde with 5% glacial acetic acid, dehydrated in an EtOH gradient, critically point dried (CPD 020; Oerlikon Balzers, Balzers, Liechtenstein) and mounted onto the tip of an insect pin using dental glue.

Nano-CT was performed for the femoral CS field of a metathoracic leg using a ZEISS Xradia 810 Ultra (Carl Zeiss X-ray Microscopy Inc., Pleasanton, CA) with a voxel size of 63 nm and a field of view of 65 µm, employing a camera binning of 1 and 1601 projections. For an overview of the femur, a metathoracic leg was imaged using a ZEISS Xradia 520 Versa (Carl Zeiss X-ray Microscopy Inc., Pleasanton, CA) with a voxel size of 0.69 µm, using a camera binning 2. The X-ray tube was operated at 40 kV and 76 µA, acquiring 1601 projections and an exposure time of 6 s per projection. The raw transmission images from both micro- and nano-scale CT imaging experiments were reconstructed using a commercial image reconstruction software package (Zeiss XMReconstructor, Carl Zeiss X-ray Microscopy Inc., Pleasanton, CA), which employs a filtered back-projection algorithm. Segmentations of the reconstructed image stacks were carried out using ITK-snap (figure 1*e*; [29]; RRID:SCR_002010) and rendered using Blender (figure 1*f*,*g*–*g*′; version 2.79; RRID:SCR_008606). Images were compiled using Corel Photo-Paint (version x6; RRID:SCR_014235).

In addition to a colour coding, we numerated the CS caps in ascending order, starting in the bottom right with 1 (top left is 11), with socket numbers corresponding to their respective caps (figure 1*f*).

### 2.2. Biomechanical analysis

We used finite-element analysis to study the deformations in a CS field induced by forces resulting from bending of the entire femur. To determine how a force applied to the entire femur affects the CS field, we applied a tensile force of 35 µN along the complete distal end of the model based on forces measured during fly take-off ([30]; arrows in figure 1*f* show force vectors). Since the femoral CS field is located on the ventral side of this leg segment, a bending of the segment upwards leads to tensile forces in this region of the segment. We distributed the tensile force evenly across all elements at the ‘face’ of the distal end of the CS model. To fix the model, its complete proximal face was constrained in all axes (trapezoids in figure 1*f*) so that an elongation of the rest of the model was still possible. Material properties of the cuticle, Young's moduli (YM; E) and Poisson's ratio (v) were taken from the measurements reported in Skordos *et al*. [22] for *Calliphora vicinia* (table 1).

**Table 1.** Material properties of CS structures and material property variations (Cases) are applied for each finite-element analysis. The YM of four structural components from *C. vicina* (CS cap, collar, socket and the cuticle). All values in GPa. Poisson's ratio for all is 0.3. Physiological values are based on [22]; their sockets are referred to as socket septum. The YM of the socket and collar used in combination (cases c-c, c-p, p-c and p-p) in this study to investigate the structural influence on strain distribution. First letter of each case is the socket YM, second letter the collar YM. ‘c’ = cuticular, ‘p’ = physiological. Arrows indicate value increase or decrease compared with physiological value. c: cuticular; p: physiological.

structure	YM				
	physiological	c-c	c-p	p-c	p-p
cap	6				
collar	4.8	1.5 ↓	4.8	1.5 ↓	4.8
socket	0.15	1.5 ↑	1.5 ↑	0.15	0.15
cuticle	1.5				

We used the open-source finite-element solver VOX-FE2 for the analysis of stress and strains in the CS field. The FE mesh and the model constraints were generated and defined using a plugin graphical user interface established in PARAVIEW (v. 4.1.0, www.paraview.org; RRID:SCR_002516, plugin from http://sourceforge.net/projects/vox-fe). The segmented geometries were exported from ITK-SNAP and converted in PARAVIEW into an FE mesh with 7 557 345 (complete), 7 511 352 (without one socket), 7 490 229 (without the other socket) or 7 127 949 (without all sockets) hexahedral elements by direct voxel conversion based on the resolution of the nano-CT.

From the finite-element analysis results, we extracted the maximum (most tensile) and minimum (most compressive) principal strains (P1/*ɛ*1 and P3/*ɛ*3, respectively) using the VOX-FE2 PARAVIEW plugin. Displacements were further analysed and visualized in R Studio (RRID:SCR_000432) and Matlab (RRID:SCR_001622).

### 2.3. Principal component analysis

**Figure 2.**
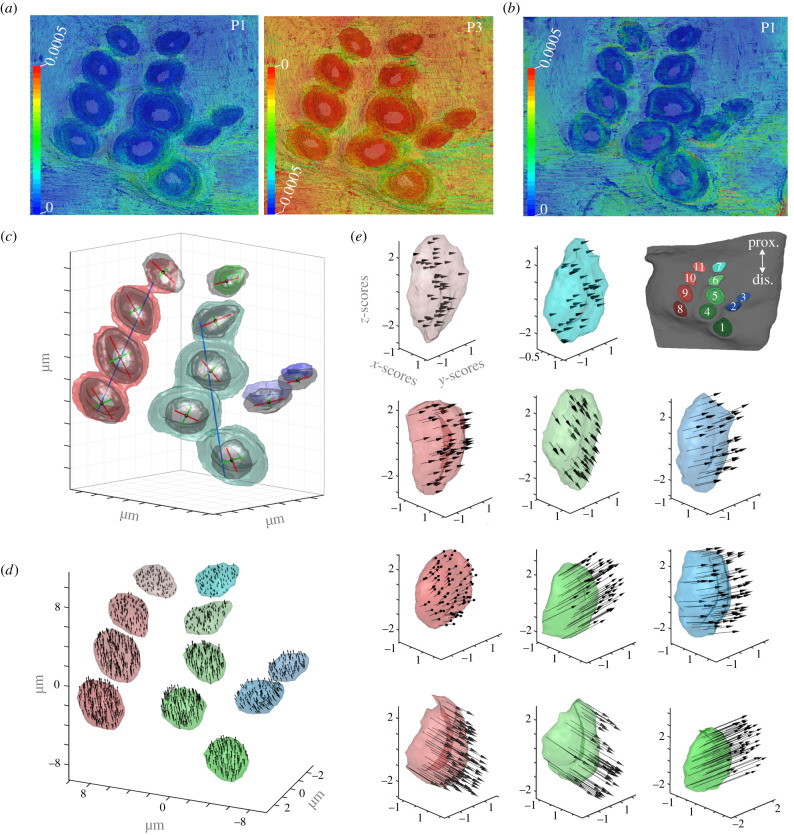
Overview of the strains, orientations and displacement directions for each CS within the femoral field of a hind leg. (*a*) Principal most tensile (P1) and most compressive (P3) strains on the outside of the leg, with view of the CS caps, during load similar to flight take-off with all structures having physiological (p) YM (case ‘p-p’: socket physiological YM, collar physiological YM); (*b*) overview of the most tensile (P1) strains in the inside of the leg, view of sockets; (*c*) the orientations of CS caps and interconnected sockets derived from the first two principal components of a principal component analysis; (*d*) displacement vectors of each cap, the arrangement of the caps corresponds to that found in FeF; (*e*) displacement vectors of each cap, the caps are oriented along their respective long axis. All positional information has been transformed from µm into normalized z-scores for a comparable depiction. Note that cap colours and locations within the figure correspond to the inset in the upper right corner of (*e*). Vectors in (*d*) and (*e*) are scaled 100-fold for legibility, only every 200th vector is displayed; prox.: proximal, dis.: distal.

We determined the main axes orientations of CS caps and their underlying sockets using principal component analysis (figure 2*c*). In other words, we used principal component analysis as a way to find the largest Cartesian dimension of CS caps. Generally, principal component analysis determines the directions of largest variance in a dataset which lie orthogonal to each other. Here, we applied it in a novel manner, to determine the directions of largest variance within the Cartesian coordinates of the surfaces generated from µCT image stacks of individual caps and connected sockets, respectively. The first two principal components describe the two main axes, (i.e. the major and minor axis of a given cap (red and green axes in figure 2*c*)). Note, this also allows for an intuitive estimate of the eccentricity of a particular cap; if the major and minor axes have similar lengths, then the cap is more circular (i.e. eccentricity is low). If the major axis is noticeably longer, the cap is more oval shaped (high eccentricity). For connected sockets, we calculated only the first principal component. Connected sockets were identified through nano-CT (figure 1*g*′). The axes (blue lines in figure 2*c*) describe the general main orientation of a particular set of connected sockets. Consequently, the third principal component gives information about the normal orientation of a given point or structure.

### 2.4. Displacement analysis

We visualized the effects of force application during finite-element analysis on individual CS caps by calculating the displacement vectors for all points between the unloaded and the loaded conditions. Because absolute displacement distances were very small relative to the average size of the CS, we multiplied the vector lengths by a factor of 100 to visualize their directions (figure 2*d*). The lengths of these arrows are equivalent to the displacement during force application. Due to the high number of elements in each finite-element analysis model, we plotted only every 200th displacement vector.

The displacement and consequent deformation of CS caps is important for understanding their function when the leg strains. To clarify further how each CS is deformed, we extracted the individual CS caps and their associated displacement vectors and re-oriented them according to their individual major and minor axes (figure 2*e*). In this depiction, the vertical axis (z) of a particular CS thereby corresponds to its major axis (identical to the red axis in figure 2*d*, first principal component), the y-axis corresponds to its minor axis (identical to the green axis in figure 2*d*, second principal component). The displacement vectors have been rotated and re-arranged correspondingly. All positional information has been transformed from micrometre into normalized z-scores for a comparable depiction.

### 2.5. Parameters

We used the same material properties as Skordos *et al*. [22] and Sun *et al*. [6] for the cap, collar, socket and cuticle (table 1). These values originate from *C. vicinia* and are phylogenetically the most comparable, available values. The lower the YM, the more flexible the material; the higher the YM, the less change in shape. In each of the four analysed cases (table 1), the YM of the socket and collar was either physiological (p) or set to the YM of the cuticle (cuticular YM; c) in order to investigate the influence of varying material parameters within substructures on overall strain patterns. By altering the YM of the structures to that of the cuticle, we could simulate the lack of a collar and sockets that react to strain in the same manner as the surrounding cuticle. To investigate how the presence of sockets influence strain distributions, we removed (i) all sockets; (ii) the socket of CS 5 or (iii) the socket of CS 9 with physiological YM parameters in all structures (case p-p; figure 1*h*–*h*′). Unlike other publications, we refer to the socket (or socket septum) and the collar as separate entities, as the morphological description of the outer morphology suggests that not all CS have collars, but all have sockets.

## 3. Results

### 3.1. Some sensilla are interconnected

The femoral field is found on the proximal, ventral end of the femur (figure 1*a,b*). The 11 CS of the femoral field are arranged in three columns and were visualized using nano-CT (figure 1*c,d,e*). Within each column, the *elliptical* CS have the same relative long axis orientation (figure 1*f*). Three CS are circular (CS 1, 4 and 5), and these are the only CS with an identifiable collar (figure 1*f*). The elliptical CS in column one (CS 8–11, left column) has a mirrored orientation to the CS in columns two (CS 6 and 7, centre column) and three (CS 2 and 3, right column; figure 1*f*).

The nano-CT reconstruction reveals physical connections between sockets 8–11 and between sockets 1 and 4–6 (figure 1*g*–*g*′). To test how these connections influence strain propagation, we removed individual sockets as shown in figure 1*h,h*′ in our finite-element analysis. Visually, the axes of the three circular CS (1, 4, 5) are similar to each other but different compared with the CS of their corresponding column. Their eccentricity is low, and the orientations of their major and minor axes should only have a weak influence on their directional sensitivity. For elliptical CS, the medial ends of their sockets have similar long axis orientations as their corresponding cap (electronic supplementary material, video S1). Interestingly, the medial ends of the sockets of the *circular* CS caps are highly *eccentric* (electronic supplementary material, video S1), and the orientation of the socket of CS 1, for example, is the same as that of the neighbouring CS sockets from elliptical caps (CS 2, 3, 4 and 5). Thus, the outer geometry of CS does not necessarily translate to their inner geometry. Additionally, the outer structures show less displacement than the internal substructures in proximal CS (CS 10–11; figure 2*a,b*), suggesting an influence of the sockets on uniform strain distribution within the field.

Finite-element analysis shows that physiological input forces similar to a flight take-off scenario, equivalent to an upward bend of the femur, translate all CS laterally (relative to the CS-centric coordinate system defined by the main axes, figure 2*c*) with higher strain in the more distal CS (figure 2*d*). The vector orientations of the resulting strain are dependent on the location of each cap (figure 2*e*). The various displacements (magnitude and direction relative to CS main axes) suggest a diverse mechanical influence throughout the field. While the more proximal CS (CS 7, 11) generally have shorter vectors, the orientation and vector length throughout columns consisting of elliptical CS with the same general long axes (e.g. CS 8–11) also show different vector lengths and orientation.

### 3.2. Campaniform sensilla substructures influence strain transduction

**Figure 3.**
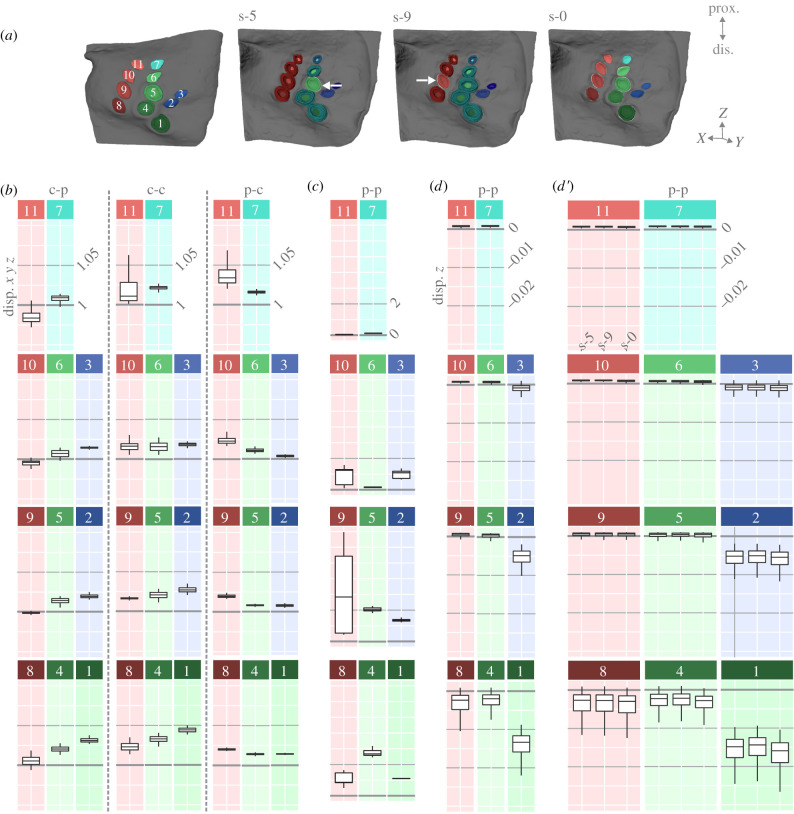
Altering YM of caps and sockets and their connections effects cap displacement. (*a*) Schematic of the caps and cases S-0, S-5, S-9; arrows indicate the removed sockets in S-5 and S-9; (*b*) relative cap displacement of caps with sockets. Boxplots show the relative cap displacement of all 11 femoral field CS for the three cases (c-p, c-c and p-c) relative to the physiological state p-p; (*c*) the relative cap displacement without sockets, note other scaling; (*d*) absolute cap displacement in the case p-p with sockets; (*d′*) absolute cap displacement with individual sockets removed (socket 5 [s-5], socket 9 [s-9]) and without all sockets (s-0). Note that box plot colours and locations in (*b*–*d*) correspond to the caps in (*a*) to aid legibility; prox.: proximal, dis.: distal.

We altered the individual YMs of the socket and collar in four different cases (table 1) to analyse their influence on CS displacement within a field. Additionally, the influence of the interconnected sockets was tested by removing all sockets or only individual sockets within a group of connected sockets (figure 3*a*). The finite-element analysis results show the highest tensile (ɛ1) and compressive (ɛ3) strains, independent of the YM cases, in the proximity of CS 1, 2, 3, 4 and 5 (figure 2*a*). Two YM cases show two opposing areas around CS 1's cap that have higher tensile or compressive displacement. This suggests that CS 1 compresses along the short axis of its socket. The other circular CS does not show similar strains (figure 2*a*).

All three material combinations with different YM compared with the physiological case (p-p; table 1) show altered displacement throughout the field. Cases c-p (socket YM higher than physiological, collar YM physiological) and c-c (socket YM higher than physiological, collar YM lower than physiological) were similar in the six most distal CS (figure 2 schematic) but differed in the displacement of the five most proximal CS. The relatively compliant collars of CS 1, 4 and 5 in c-c reduced the displacement of proximal CS, whereas physiologically elastic collars in the case c-p led to more displacement in CS 9, 10 and 11. In general, harder collars affected displacement in proximal CS (figure 3*b*).

When the sockets were harder (c-c), the effect on cap displacement was reversed relative to the previous experiments (p-c, figure 3*b*). The caps that displaced less than in the physiological case (p-p) when the socket was harder (CS 10, 11) displaced more when the socket was physiological (c-c). Additionally, in c-p, where the sockets are harder than physiological and the collar is physiological, the only displacements that decrease are those of CS 9–11 (table 2). Caps that displaced more with a cuticular socket than in p-p (caps 1, 2, 3, 4 and 5) displaced less when the socket was physiological (p-c, socket physiological YM, collar lower than physiological YM). Thus, harder sockets reduced tensile strains in distal CS while increasing tensile strains in proximal CS (for arrangement figure 1*f*). Ultimately, this material correlation allows a more uniform strain distribution throughout the field, possibly providing more CS stimulation overall.

**Table 2.** Cap displacement during modulation of substructure YM compared with the physiological state. Summary of results from figure 3*b*. Effects on cap displacement relative to the physiological case. Differences indicated through arrows, either indicating increase (upwards arrow) or decrease (downwards arrow). The arrow location represents the location of the CS cap, i.e. the top row arrows are CS 11 and 7; the second row are CS 10, 6, 3; the third row CS 9, 5, 2; fourth row CS 8, 4, 1.

cuticular socket YM, physiological collar YM (c-p)			cuticular socket YM, cuticular collar YM (c-c)			physiological socket YM, cuticular collar (p-c)		
↓	↑		↑	↑		↑	↑	
↓	↑	↑	↑	↑	↑	↑	↑	↑
↓	↑	↑	↑	↑	↑	↑	↑	↑
↑	↑	↑	↑	↑	↑	↑	↑	↑

When all sockets were removed (s-0; figure 3*a,c*), there was an overall increase in displacement throughout the field that affected all CS except the two most proximal (CS 11 and 7). This displacement increase works in a gradient, most affecting the second most distal CS of each column (CS 4 and 9) and affecting the third most distal CS (CS 10 and 5) to a lesser degree (for CS arrangement figure 1*f*). This indicates that sockets affect the overall compliance of the field.

The absolute displacement (figure 3*d–d′*) shows compression in the five distal CS (1, 2, 3, 4 and 7) when physiological parameters were used. When all sockets were removed (s-0; figure 3*a,d*′), a minor increase in compression could be seen in the aforementioned CS. Removing single sockets of those that are interconnected did not significantly affect the absolute displacement, but did affect the overall displacement in comparison to the physiological (p-p) condition with sockets (figure 3*a,d*′). This indicates that sockets can alter the displacement of CS caps, underlining that socket networks have a function in the transduction mechanism.

### 3.3. Socket properties affect strain levels in caps and collars

**Figure 4.**
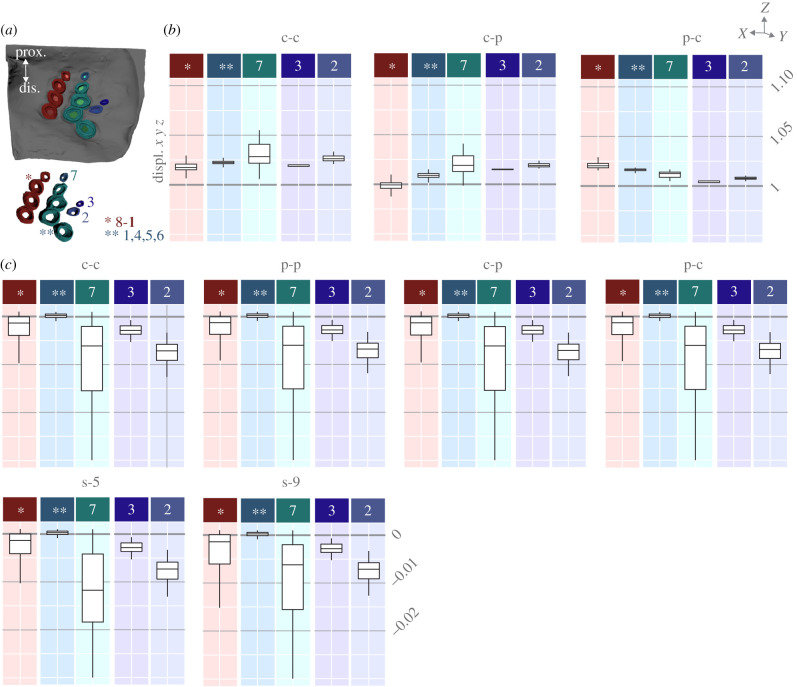
Group-wise displacements when altering YM of caps and sockets. (*a*) Schematic of the numerated sockets; (*b*) Relative socket displacement in the cases c-c, c-p, p-c relative to p-p; (*c*) absolute socket displacement in the three cases, s-0, s-5 and s-9. Note that box plot colours and locations in (*b*) and (*c*) correspond to the sockets in (*a*) to aid legibility; prox.: proximal, dis.: distal.

We analysed the effect of altering the YM on the socket (figure 4*a*) displacement in the same manner as the cap. The greatest tensile strain can be seen around the distal-most sockets and the socket of CS 11. All YM cases caused either an increased displacement or no change (figure 4*b*). Harder sockets (figure 4*b*, c-c) tended to displace more than physiological sockets (figure 4*b*, c-p). Sockets that are interconnected displaced less when the sockets were harder and the collars were more compliant (figure 4*b*, c-c). Physiological sockets with more compliant collars affected the unconnected sockets more by decreasing their displacement compared with the other cases (figure 4*b*, p-c).

As shown previously, the caps of CS 9, 10 and 11 displaced less when the socket was harder than physiological (c-p) (figure 4*b*). When the collar was harder (c-p), it caused more compressive strain in the socket (figure 4*b*). The entire column (CS 11, 10, 9 and 8), in turn, displaced the most when the socket was more compliant than physiological (p-c) (figure 4*b*). When the socket YM was physiological, there was the most tensile strain compared with the other cases (figure 4*b*). This indicates that a more compliant socket increases tensile cap strain, whereas a harder socket increases cap compression.

Predominantly, the sockets that are not interconnected (CS 2, 3 and 7) showed the greatest compression (figure 4*c*). The connected sockets of CS 8–11 also compress but to a lesser degree. The interconnected sockets of CS 1, 4, 5 and 6 did not compress in any case (figure 4*c*). Generally, altered YM parameters had little effect on the absolute displacement of the sockets, and both connected and unconnected sockets responded to changes in compliance. The absolute displacement of the sockets (figure 4*c*) shows that there are compressive strains throughout the network, except in the socket of CS 7. Again, there were no clear differences between the YM cases or through the removal of individual sockets.

## 4. Discussion

The application of physiological input forces on a highly detailed CS field shows that the material properties and the specific arrangement of collars and sockets affect the displacement of caps throughout the field. Moreover, our results demonstrate that circular CS could indeed have directional sensitivity based on the geometry of their collars and that sockets are partially interconnected in groups.

We studied the displacement of the femoral CS field during the application of physiological strain. The field contains sensors with differences in all key morphological aspects (orientations, eccentricity, collared/uncollared; figure 1*f*). The combination of these different CS morphologies in one field may influence the sensitivity of individual sensors, as the relationship between and the shape of cuticular holes can tune the rate of change in strain [21]. We therefore used this field to investigate how physical connections between CS as well as different physical CS parameters affect the displacement throughout the field upon the application of tensile loads that mimic typically occurring forces and force directions. By altering the YM of CS subunits or removing these structures altogether, we analysed the roles of different CS components.

Our results show that CS sockets within the femoral field are partially interconnected and that this influences strain distribution. Generally, the sockets seem to be displaceable entities with their own directional sensitivity, and they transform the stimulus along their axes (figure 3). Unlike the findings reported in Skordos *et al*. [22], our data underline that the stiffness of the sockets can affect CS displacement. Additionally, although collars only surround the three circular CS, they influence the displacement of the entire field (figures 2*d* and 3*b*). This underlines that major structural components of CS, sockets and collars regulate the amplification of strain.

### 4.1. Caps

Grouping CS based on the orientation of their long axes is suggested to unitize their functionality as they will respond to similar forces [1,25,31]. All eight elliptical CS in the femoral field are orientated at roughly 45° to the long axis of the limb (figures 1*f* and 2*c*). This suggests that they are most sensitive to torques, as torques produce helical compressions and tensions that are maximal along the line oriented 45° to the leg's long axis [11]. Elliptical CS oriented either perpendicular or parallel to the leg's long axis, on the other hand, would respond to ventral or dorsal bending, respectively [11]. The presence of different long axis orientations within one field suggests that it is exposed to directionally antagonistic strains. Other insects, such as *C. morosus*, have multiple CS in spatially separated groups on the femur, with each group having one dominant CS orientation, and these groups together build one field [32]. Together, they encode forces of the coxo-trochanteral joint and forces that produce a twisting motion in the femur. These twisting forces may occur during stepping movements [33]. It is possible that in the smaller femur of *D. melanogaster*, one field of CS with different directional sensitivities encodes all relevant strains for the motor output control during posture and locomotion.

In addition to orientation variability, differences in CS cap sizes are present ([25], figure 2*c*). In our study, we found a size gradient in each column, with the smallest CS in the most proximal positions and the largest in the most distal positions (figures 1*f* and 2*c*). This cap size gradient is also represented through gradients in displacement strength (figure 3). Cap size, as well as elastic properties, can modulate CS sensitivity [17,24,34]. The cap size gradients may thus divide strain into narrow ranges to allow for a more precise representation of strain levels during different locomotion tasks.

Encoding of directional strain is commonly associated with elliptical CS [11]. The circular CS (1, 4 and 5) may be omnidirectionally responsive or directionally sensitive if their caps are asymmetrically located within their collar [10,35]. CS 4 and 5 appear evenly surrounded by their collars and may function as tactile receptors that respond equally well to stresses applied from any direction [15]. CS 1, on the other hand, is asymmetrically located within its collar, which ultimately provides this CS with axes that may lead to directional sensitivity (figures 1*f* and 2*a*) [36]. However, Dinges *et al*. [16] showed that the relative location of this CS is variable between individuals, and the asymmetry seen here may not be representative of all femoral fields.

### 4.2. Sockets and collars

Through altering the YM of CS substructures, we investigated their effects on a CS field's dynamics. The YM of the collar could influence the displacement of an individual CS cap; however, the socket, cuticular cap and spongy cuticle YM do not affect the overall coupling mechanism (the lateral displacement of the cap induced by cuticular compression; [22]). Based on our data, we propose that socket properties are relevant for overall displacement within a network, as both more compliant and harder sockets change the displacement. Some CS displace less with harder sockets while others displace more (figure 3*b*). This is apparent when the collar's YM is physiological and the sockets' YM is increased (c-p), where the proximal CS displace less than the distal, and the right column CS displace more than the left. Thus, connected sockets may play a functional role and may have their own compressional axes, making them susceptible to strain stimuli.

Sockets have been described as a part of the stimulus conversion during which the dendritic tip, which is surrounded by the socket, is transversely compressed and the cap is displaced vertically [22,37]. In hair shafts, which undergo a similar lateral displacement, the dendrite's movement is restricted by the resistance and stability of the socket during compression [38,39]. It is also predicted that the medial socket end may add a factor of directional sensitivity by limiting the movement of the dendrite [38]. Our data suggest that, in addition to the relevance of sockets for individual CS, the socket network influences the entire field. There is a gradual distribution of strain through the sockets in the CS that are united by connected sockets (figure 3). The strain in our model was applied along the long axis of the femur (figure 1*f*), but the finite-element analysis results show the strain distribution on a diagonal aligning with the sockets. Thus, the morphology of the interconnected sockets alters the proliferation of strains along the cuticle.

For a single CS, a 30% change in collar stiffness can amplify the cap displacement 1.5-fold [22]. Our data show that within this CS field, the collars of a single CS can also affect the displacement of multiple CS (figure 3). Furthermore, as no collars were identified around the elliptical CS, they may be less sensitive to strain as they do not have a stiff surrounding ring for signal amplification.

### 4.3. Context integration

The femoral field can be compressed during behaviours such as walking and jumping through the resistance of depressor muscle activity [25]. When the depressor is active, the femur presses down toward the ground, and this force is resisted by the substrate, which causes bending of the femur. Our simulation shows that this dorsal bending of the femur indeed displaces femoral field CS, most prominently the distal CS.

To improve the model in the future, the material properties for *D. melanogaster* CS should be measured using nano-indentation [40]. Skordos *et al*. [22] depicted that homogeneous material properties can negatively affect physical CS models, with directionally opposite cap movements based on the homo- and heterogeneity of the cap as well as differences in output magnitude. Thus, future models should implement the anisotropic viscoelastic material properties which are most likely also characteristic for CS fields and their surrounding cuticle. Further, in other insects, like a mantid (Mantidae) and a beetle (Tenebrionidae), it has been shown that joint stiffness varies throughout the leg, which could have considerable effects on the nearby CS [41].

It is difficult to directly correlate strain patterns and magnitudes with CS activation patterns at this stage, as the neuronal encoding of cap compression has yet to be established. Moran *et al*. [7] used cockroaches to estimate that a CS cap needs to be compressed by 10 to 20 nm for the sensory neuron to fire, but other studies suggest that a displacement of 1 nm may be sufficient [21]. In future experiments, the effect on CS displacement through a femoral movement should be correlated with CS signalling through, for example, mechanical displacement of the femur during calcium imaging of the femoral field.

Our results contribute to our understanding of force-to-strain transformation in distinct CS locations. By expanding the acquired CS locations and limb displacement directions, as well as studying larger insects with different CS morphologies, we will be able to investigate how natural limb movements produce different strain portfolios, ultimately affecting CS stimuli. Furthermore, the interindividual variability in CS fields is likely to change strain amplification and strain exposure between individuals [16]. Comparing finite-element analysis from multiple individuals' CS locations will give us insight into CS function and the nervous system's role in equalling out morphological differences.

## 5. Conclusion

The application of physiological input forces on a highly detailed CS field model shows that the properties of collars and sockets affect the displacement of caps throughout the field. Moreover, sockets are structurally interconnected within a field, creating functional subunits that alter the strain distributions. Our results further demonstrate that circular CS could indeed have directional sensitivity based on the geometry of their collars.

The interconnectedness of the nervous and biomechanical systems cannot be ignored, as CS consist of mechanical pathways that link extracellular structures to neuronal signalling pathways [6]. Future studies will need to consider how CS arranged in groups/fields, with their particular substructure arrangement, encode the dynamics of cuticular strains for the neuronal system.

## Data Availability

Data have been made available https://doi.org/10.5281/zenodo.5960143.
